# Computational Biology in Colombia

**DOI:** 10.1371/journal.pcbi.1000535

**Published:** 2009-10-30

**Authors:** Silvia Restrepo, Andrés Pinzón, Luis Miguel Rodríguez-R, Roberto Sierra, Alejandro Grajales, Adriana Bernal, Emiliano Barreto, Pedro Moreno, Maria Mercedes Zambrano, Marco Cristancho, Andrés González, Harold Castro

**Affiliations:** 1 Laboratorio de Micología y Fitopatología, Departamento de Ciencias biológicas, Universidad de los Andes, Bogotá, Colombia; 2 Centro de Bioinformática-Instituto de Biotecnología (IBUN), Universidad Nacional de Colombia, Bogotá, Colombia; 3 Grupo de Investigación en Bioinformática, Escuela de Ingeniería de Sistemas y Computación, Universidad del Valle, Cali, Colombia; 4 GEBIX- Centro Colombiano de Genómica y Bioinformática, Bogotá, Colombia; 5 CENICAFE-National Center for Coffee Research, Km 4 vía a Manizales, Chinchiná, Caldas, Colombia; 6 Grupo de Diseño de Productos y Procesos (GDPP), Departamento de Ingeniería química, Universidad de los Andes, Bogotá, Colombia; 7 Grupo de Comunicaciones y Tecnología de Información, Departamento de Ingeniería de sistemas, Universidad de los Andes, Bogotá, Colombia; University of California San Diego, United States of America

High-throughput techniques are somewhat restricted in developing countries. However, computational resources have evolved in recent years to become available to the general public, with greater ability to solve intense computational problems at low cost. Therefore, the vast amount of information that is currently being generated and the need for finding the underpinnings of several issues in biology, have been the impetus of the computational biology area in Latin America. Colombia is no exception, as its rich genetic diversity has convened the attention of several institutions, including both governmental and academic departments, to find how, where, and when these resources could be employed to its benefit. In this review, we introduce the efforts being made throughout the country to spread the word and establish a strong network from a mid- and long-term perspective.

In Colombia, computational biology is just starting to be known as a field of research in its own right. Until now, mainly chemists and biologists have used bioinformatics as a tool to try to solve their particular research problems. We start by reviewing the work of some research groups and their projects. Next, we identify the driving forces of computational research and the problems to be faced in the future. This review is not exhaustive and we apologize for any groups that were not mentioned or acknowledged. (See Box 1 for Authors' Biographies.)

**Box 1 Authors' Biographies**

**Silvia Restrepo** was formerly a post-doctoral associate at Cornell University and is currently an associate professor at Universidad de los Andes. She teaches courses in Bioinformatics, Biology of the Fungi, and Phytopathology. Silvia has obtained several grants from the Colombian Ministry of Agriculture and is currently an advisor for many students at the University. She pioneered bioinformatics research in Colombia with innovative studies applied to agriculture. **Andrés Pinzón** is a doctoral student and bioinformatician at Laboratorio de Micología y Fitopatología (LAMFU), Universidad de los Andes. **Luis M. Rodríguez-R** is a Master's student and bioinformatician at LAMFU; he is also a bioinformatician for the GeBiX project. **Roberto Sierra** did his Master's studies at LAMFU and is now a research assistant. Roberto also works for the GeBiX project analyzing metagenomic data from 454 sequencing. **Alejandro Grajales** was formerly a research assistant at LAMFU and was recently accepted as a doctoral student at the American Museum of Natural History in New York. **Adriana Bernal** is an associate professor at Universidad de los Andes and leads LAMFU with Silvia Restrepo. **Emiliano Barreto** is a professor at Universidad Nacional de Colombia. **Pedro Moreno** is a professor at Universidad del Valle. **María Mercedes Zambrano** is the scientific director of Corpogen and the leader of GeBiX project. **Marco Cristancho** is the leader of the bioinformatics unit at Cenicafé. **Andrés González** is a professor at the chemical engineering department at Universidad de los Andes. **Harold Castro** is an associate professor at the Systems Engineering Department at Universidad de los Andes and a pioneer in grid computing in Colombia.

## Research

In Colombia, research in computational biology started in a group from pharmaceutical chemistry at Universidad Nacional de Colombia. CBIB, as it is known, was formed in 1998 and has since been headed by Emiliano Barreto. This group has been the Colombian node of the EMBnet (European Molecular Biology network) since 2002 and has been a member of the Ibero-American network of bioinformatics since 2004. It has developed computational biology for research, teaching, and university extension-course purposes. Its participation in educational activities is noteworthy (see below), due to a collaboration with the Swiss Institute of Bioinformatics. It has also developed bioinformatics tools for the storage, handling and analysis of biological data.

For a long time, the group has focused on the study of the beta-lactamases, particularly the description of these enzymes and a general understanding of resistance to beta-lactamic antibiotics. Two developments in this area are BLEE, (http://bioinf.ibun.unal.edu.co/servicios/BLEE/) and BLA.id (http://bioinf.ibun.unal.edu.co/BLA.id/). BLEE was designed by the CBIB in order to organize and systematize information related to an expanded spectrum of beta-lactamases. The BLEE system consists of three modules: (1) an introduction to the basic theory behind the study of the enzymes; (2) a description of the most important biochemical and physico-chemical properties of this group of molecules, as well as the different families they belong to (SHV, CTX-M, OXA, TEM); and (3) a Website where users can find an online tool developed by CBIB to identify families of an extended spectrum of beta-lactamase(s) by their biochemical properties. BLA.id is an information system that allows for molecular and clinical data-crossing, making possible identification of beta-lactamases from resistant organisms at the intra-hospital level on the basis of their sequences. BLA.id allows users to: analyze sequences; identify them by class, family, or variant levels; build dendrograms; and search/browse related information such as manual and automatic annotation, comments, bibliography, geographical references, alternative names, and source organisms.

Plant pathology has been a key area for the development of computational biology in Colombia because of the country's commitment to agriculture, with plant pathology and the breeding of crops being a national priority. Coffee has historically been the main agricultural export product of Colombia, accounting for 17% of the agricultural production in 2008, and giving employment to more than 560,000 coffee-growing families [1]. The National Center for Coffee Research (Cenicafé) is in charge of coffee research undertaken by the country; the center has developed a resource-rich platform for the storage and analysis of genomic data gathered since 2000 [2]. Its aim is to develop tools for biologists so they can access and process the genomic information without prior knowledge of programming or database development. They have succeeded in organizing the molecular data collected in coffee and other organisms as structural databases that can be accessed on the Web (http://bioinformatics.cenicafe.org/). Initially, they constructed ESTs (expressed sequence tag) and microsatellite coffee databases by means of specialized interfaces that allowed users to search, update, delete, and insert text data, sequences, images, and documents. Their platform, which also includes an in-house–developed LIMS (laboratory information management system), can also be accessed at this same Web address. Moreover, the group maintains a strong collaboration with the Sol Genomics Network (SGN) as the mirror of their site in Colombia. Besides building a very robust platform for research on coffee, Cenicafé has performed such important advances as coffee genomics [3]–[5], coffee genetic diversity studies [6], the understanding of plant resistance to diseases and pests [7], the molecular study of coffee pathogens [8]–[10], and the diversity of *Beauveria bassiana* [11], a fungus with biological control potential not only for coffee pests, but for a wide spectrum of other plant diseases in Colombia.

At the Universidad de los Andes, the mycology and plant pathology laboratory (LAMFU), led by Adriana Bernal and Silvia Restrepo, maintains an active field of computational biology research in to two diseases of crops relevant to third world countries: potato and cassava. On one hand, the group had been studying the oomycete pathogen *Phytophthora infestans*, a destructive pathogen of potato crops. The study of this pathogen's genome has led to the development of bioinformatics and molecular tools that are useful in characterizing this oomycete's populations [12],[13]. The group is also collaborating on the annotation of the *Phytophthora infestans* genome [14]. On the other hand, they have sequenced the complete genome of the cassava pathogen *Xanthomonas axonopodis* pv. *manihotis*, and have become one of the teams participating in the annotation of three *Xanthomonas* genomes. LAMFU's Web site (http://bioinf.uniandes.edu.co/) allows access to and exploration of the microsatellite database, PhytoSSR, of three *Phytophthora* species [12] and displays some tools simultaneously developed with the analysis of the genomes. For example, BLAST-it is a bash shell script that performs multiple BLAST alignments against a predefined BLAST database; also available is BLAST-it4web, where the user can create their “own” BLAST database “on the fly”. This script is useful when there exists a batch of FastA files and users with limited access to computational resources need to perform a BLAST comparison for each sequence against a given database. LAMFU also developed Amico, a script that identifies a given amino acid composition on the first “n” residues of an amino acid FastA (single/multiple) sequence file. This Web site is commonly used by Colombian molecular biologists for teaching and for program and algorithm testing. Finally, in an effort to understand the molecular interaction between potato plants and *P. infestans*, the team successfully reconstructed the metabolomics of the diseased potato by a systems biology approach based on our own microarrays data (unpublished results and [15]).

Located at Universidad del Valle in Cali, Colombia, Pedro Moreno has developed a scalar theory that tries to explain population repertoires of cells during humoral immune response [16]. Also, his group developed a novel and multidisciplinary approach to study DNA sequences based on the representation of chaos theory using multifractal analysis, thanks to the collaboration with engineers. Nowadays they are collaborating with a bioinformatics group at Universidad de Cauca led by Patricia Vélez that is trying to identify some biological problems that might be solved with computational tools. In particular, they are focusing on molecular, mathematical, geometrical, and statistical bases of the structural and functional organization of genes, genomes, proteomes, and interactomes. By utilizing approaches derived from bioinformatics, fractal geometry, linguistics, systems biology, and computational sciences, they are developing algorithms and tools designed to find answers about properties that characterize life.

The systems biology team inside the Grupo de Diseño de Productos y Procesos (GDPP) at Universidad de los Andes, led by Andrés González, is applying systems biology tools to product design and biological system optimization from an industrial perspective. For example, they are assessing gene expression noise in quorum-sensing–regulated gene networks by stochastic modeling. Once the evolution of the signal in time is obtained, they can introduce optimization strategies to find adequate doses of quenchers to minimize signal concentration, and hence the pathogenic phenotype fails to evolve in the host cell. These algorithms have been applied to *Pseudomonas aeruginosa* biofilm removal from abiotic surfaces with interesting results [17]. Moreover, they are studying the potentially bistable response in *Bacillus thuringiensis* using bifurcation analysis. Based on a deterministic model, they found that *B. thuringiensis* could also exhibit a bistable response during sporulation, and that the transition from planktonic to sporulated state is strongly influenced by noise within the cell. Interestingly, they proposed a novel scale-down in silico approach to determine cellular reactions to periodic variations in oxygen tension by analyzing the effects of amplitude and frequency of cells on the distribution of the population and protein frequency based on Fast Fourier Transform analysis [18]. Taking into consideration the need for sustainable technologies for energy generation, González and his team are applying systems biology tools to optimize the performance of microbial fuel cells and to engineer bacteria for the synthesis of alcohols based on glycerol. They are currently developing a bi-level platform based on a genomic scale model of *P. aeruginosa* that is capable of predicting deletions or overexpression of genes in order to optimize the production of the electron shuttle molecules and therefore increase power density [19]. This platform will be extrapolated later to optimize the production of ethanol in *Escherichia coli* without external electron acceptors using a similar genome-scale model. Rational product design is also being carried out in his lab with a systems engineering approach. In particular, the team, in collaboration with LAMFU, is developing algorithms that can predict mutations in specific lipases that degrade the quorum-sensing signal of the commonly known plant pathogen *Ralstonia solanacearum.* These algorithms are built on ab initio protein models of molecular mechanics and dynamics [20].

The group at the Universidad del Valle is not the only team collaborating with engineers. At Universidad de los Andes, biologists work with systems and computer engineers to build a robust computational infrastructure. Harold Castro's group is designing and implementing a grid infrastructure that utilizes the entire computational capacity of the University to help solve scientific problems. It includes not only dedicated clusters, but also computer rooms scattered across the campus. This solution is based on virtualization and allows researchers to use both opportunistic and dedicated resources, with great independence of the platform. Users build an image of their machine (operating system, libraries, applications, etc.) and deploy a virtual cluster on the physical machines available or required. This strategy allows researchers to work on the platform of their choice while taking advantage of resources provided by any collaboration grid in which the institution participates. Actually, the *B. thuringiensis* deterministic model could be built using the grid infrastructure.

Nowadays, Colombian researchers are studying Colombian biological diversity as a means of responding to worldwide concern about the loss of diversity due to global climate change and the need for alternative sources of energy. Diversity is explored at different levels. The CBIB group at Universidad Nacional created ENKI-DB [21], a database that maintains a complete repertoire of all the taxonomic (species) and molecular (DNA, proteins) information of Colombian species that are present in the most important databases worldwide. ENKI is a project financed by Colciencias and realized with the collaboration of the Instituto de Ciencias Naturales and the Instituto Von Humboldt, institutions created to promote, coordinate, and perform research that contributes to the knowledge, conservation, and use of biodiversity as a tool of development and welfare of Colombian citizens. The ENKI database also offers BLAST ENKI, an adaptation of BLAST to perform comparisons against databases of Colombian species. The taxonomic information contained in ENKI is updated on a daily basis, thanks to its connection with SIB (Sistema de Información sobre Biodiversidad [biodiversity information system]). Today the system contains 33,800 entries of Colombian species.

Two metagenomic efforts are being conducted in Colombia. The first, conducted by a consortium of institutes and universities, is exploring the microbiota present in potato-cultivated soils. For this purpose, CBIB created software to design probes for a microarray that can determine the presence of microorganisms and their metabolic activities in cultivated soils. A second metagenomics project aims to study the microbiota present in soils and water reservoirs of a national park (Parque Natural Nacional de los Nevados). This program is being conducted by GeBiX, the Colombian Center for Genomics and Bioinformatics of Extreme Environments (http://www.gebix.org.co). GeBiX, in collaboration with LAMFU, is building and implementing pipelines for the analysis of the metagenomics molecular data. The most remarkable feature of GeBiX is that it constitutes the first effort to bring together most of the main players in computational biology in the country to establish a nationwide platform for research in bioinformatics.

We have initiated a wiki Website at http://bioinf.uniandes.edu.co/colombia/ to facilitate the creation and editing of profiles of research groups in computational biology in Colombia. We invite researchers to register themselves and their groups to gather the information of all Colombian scientists involved in bioinformatics and computational biology research. This wiki could be a seed for the creation of a computational biology society in Colombia.

## Educational activities

In Colombia, there is a lack of bioinformatics courses in general, as well as more specialized courses in computational biology. The high demand for extension courses is evidence of this deficiency. The CBIB group at Universidad Nacional de Colombia has organized nine extension courses in collaboration with the Swiss Institute of Bioinformatics. Topics have varied from an introduction to bioinformatics to microarray analyses. Universidad de los Andes and Universidad Nacional are among the few universities to offer regular undergraduate and graduate bioinformatics courses. Pontificia Universidad Javeriana is the only university to offer a minor in bioinformatics and computational biology for a Master's in biological sciences. Furthermore, Universidad de los Andes has one of the few Chemical Engineering departments that offers a systems biology course for undergraduate and graduate students.

Cenicafé has also offered several bioinformatics training courses, organized at its headquarters with instructors drawn from research partners such as the former TIGR (now the J. Craig Venter Institute), Cornell University, and the University of Arizona. Scientists from a broad spectrum of Colombian research centers and universities have participated in these courses.

In an effort to expand the field of bioinformatics in Colombia, the Universidad de los Andes developed a virtual learning environment (VLE) in the bioinformatics field. The VLE is called Bioinfórmate and can be accessed at http://bioinformate.uniandes.edu.co.

## Conclusions and perspectives

Investment in overall research and development in Colombia is not large and is ranked below many other Latin American countries [22]. However, there are several private and public research centers and universities that make an effort to carry out research, especially in the agricultural sector. And they have participated in a positive manner in the development of genomics and bioinformatics in recent times. However, we believe that bioinformatics and computational biology can be bolstered in Colombia by:

- The implementation of governmental policies to support interdisciplinary courses and graduate degrees in universities around the country to train young scientists in computational biology;
- The establishment of special funding by local funding agencies or agreements with the information technology and communication (ITC) industry to support bioinformatics research;
- The development of a national grid for the analysis and sharing of bioinformatics tools and genomic data as well as improvements to the current computational capacity;
- The foundation of a Colombian national society in computational biology and bioinformatics that can organize annual meetings, gather information, and identify people currently working in computational biology, among other activities.

Many challenges lie ahead for Colombian scientists. Apart from continual efforts needed to study, analyze, and catalog its notable genetic diversity, agricultural research must be sustained as a key area of investment for improving the productivity and competitiveness of Colombia's agricultural sector [22]. Other areas where Colombian scientists can play a crucial role are climate change and its consequences, and the emergence of new human, animal, and plant diseases. Global climate change is already affecting agricultural practices in the country, as witnessed in a new spectrum of coffee diseases that are already becoming evident [23].

## References

[1] MADR (Ministry of Agriculture and Rdural Development) (2009). Estadísticas sector agropecuario.. http://www.minagricultura.gov.co/archivos/estadistica_agropecuarias_11_junio_2009.pdf.

[2] Cristancho MA, Gaitán A, Rivera LF, Orozco CA, Chalarca A (2008). Sistema de información para el manejo de datos moleculares en café: 1. Desarrollo y uso de herramientas.. Rev Acad Colomb Cienc.

[3] López G, Cortina H, McCouch S, Moncada M (2009). Analysis of genetic structure in a sample of coffee (*Coffea arabica* L.) using fluorescent SSR markers.. Tree Genet Genomes.

[4] Moncada P, McCouch S (2004). Simple sequence repeat diversity in diploid and tetraploid *Coffea* species.. Genome.

[5] Cristancho MA, Gaitán A (2008). Isolation, Characterization and Amplification of Simple Sequence Repeat Loci in Coffee.. Crop Breed Appl Biotechnol.

[6] Chaparro A, Cristancho MA, Gaitán AL, Cortina H (2004). Genetic diversity of *Coffea arabica* L. from Ethiopia using RAPD markers.. Genet Resour Crop Evol.

[7] Herrera J, Alvarado G, Cortina H, Combes M, Romero G (2009). Genetic analysis of partial resistance to coffee leaf rust (*Hemileia vastatrix* Berk & Br.) introgressed into the cultivated *Coffea arabica* L. from the diploid *C. canephora* species.. Euphytica.

[8] Cristancho MA, Escobar C (2008). Transferability of SSRs markers from related Uredinales species to the coffee rust (*Hemileia vastatrix*).. Genet Mol Res.

[9] Galvis CA, Leguizamón JE, Gaitán ÁL, Mejía JF, Álvarez E (2007). Detection and identification of a 16SrIII-related phytoplasma associated with coffee crispiness disease in Colombia.. Plant Dis.

[10] Marin M, Castro B, Gaitan A, Preisig O, Wingfield B, Wingfield MJ (2003). Relationships of *Ceratocystis fimbriata* isolates from Colombian Coffee-Growing Regions Based on Molecular Data and Pathogenicity.. J Phytopathol.

[11] Gaitan A, Valderrama AM, Saldarriaga G, Velez P, Bustillo A (2002). Genetic variability of *Beauveria bassiana* associated with the Coffee Berry Borer *Hypothenemus hampei* and other insects.. Mycol Res.

[12] Garnica DP, Pinzon AM, Quesada-Ocampo LM, Bernal AJ, Barreto E (2006). Survey and analysis of microsatellites from transcript sequences in *Phytophthora* species: frequency, distribution, and potential as markers for the genus.. BMC Genomics.

[13] Vargas AM, Quesada-Ocampo LM, Céspedes MC, Carreño N, Gonzalez A (2009). Characterization of *Phytophthora infestans* populations in Colombia: first report of the A2 mating type.. Phytopathology.

[14] Haas BJ, Kamoun S, Zody MC, Jiang RHY, Handsaker RE (2009). Genome sequence and comparative analysis of the Irish potato famine pathogen *Phytophthora infestans*.. Nature.

[15] Restrepo S, Myers K, del Pozo O, Martin GB, Hart A (2005). Gene profiling of a compatible interaction between *Phytophthora infestans* and *Solanum tuberosum* suggests a role for carbonic anhydrase.. Mol Plant Microbe Interact.

[16] Burgos JD, Moreno-Tovar P (1996). Zipf scaling behavior in the immune system.. BioSystem.

[17] González AF, Covo V, Medina LM, Vives-Florez M, Achenie L (2008). Quorum quenching analysis in *Pseudomonas aeruginosa* and *Escherichia coli*: network topology and inhibition mechanism effect on the optimized inhibitor dose.. Bioprocess and Biosyst Eng.

[18] Villamizar M, Cuervo N, Lozano G, Orduz S, Restrepo S (2009). Mesoscale Modeling of the *Bacillus thuringiensis* sporulation network based on stochastic kinetics and its application for in silico scale-down..

[19] Mejía J, Racines F, Velasco N, Cortes M, Vives-Florez M (2009). Genome Scale Modeling of *Pseudomonas aeruginosa* in a microbial air-cathode single chamber fuel cell..

[20] Alvarez A, Bernal A, González Barrios AF (2009). Virtual Screening of Quenchers for the Signal 3-Hydroxypalmitic Acid Methyl Ester in *Ralstonia solanacearum*..

[21] Pinzón A, Reguero M, Barreto E (2006). ENKI-DB: Molecular and taxonomic data integration system for Colombian species.. Rev Colomb Biotecnol.

[22] Stads GJ, Romano L (2008). Colombia. ASTI Country Brief No. 39.

[23] Cristancho MA, Escobar C, Ocampo JD (2007). Evolution of races of *Hemileia vastatrix* in Colombia.. Cenicafé.

