# Correction: Computational Biology in Colombia

**DOI:** 10.1371/annotation/35cba0b0-056a-4dd9-9b67-680d4bc69243

**Published:** 2009-11-04

**Authors:** Silvia Restrepo, Andrés Pinzón, Luis Miguel Rodríguez-R, Roberto Sierra, Alejandro Grajales, Adriana Bernal, Emiliano Barreto, Pedro A. Moreno, Maria Mercedes Zambrano, Marco Cristancho, Andrés González, Harold Castro

The eighth author's name was incomplete. The correct name is: Pedro A. Moreno.

